# Nail-Retaining Derotation Osteotomy for Subtrochanteric Fracture Malunion: A Case Report and Review of the Literature

**DOI:** 10.7759/cureus.95506

**Published:** 2025-10-27

**Authors:** Ioannis G Spyrou, Meletis Rozis, Iordanis Varsamos, Spyros G Pneumaticos

**Affiliations:** 1 3rd Department of Orthopedic Surgery, National and Kapodistrian University of Athens, KAT General Hospital, Athens, GRC

**Keywords:** antegrade intramedullary nail, derotation osteotomy, femoral anteversion, femoral malrotation, subtrochanteric fractures

## Abstract

Rotational malalignment is relatively common after intramedullary nailing of femoral fractures and may lead to significant clinical symptoms. Comminution, inherent differences in femoral version, and the absence of reliable clinical markers make achieving anatomical rotational reduction challenging, even for experienced surgeons. Various methods have been described to surgically correct rotational deformities, depending on the time elapsed since the original surgery and whether the fracture has already united. Both open and closed techniques have been used, along with different types of osteosynthesis hardware. We present a case of a subtrochanteric fracture malreduction fixed in 30° of internal rotation, which was corrected through an open femoral osteotomy using a Gigli saw while retaining the original nail three months after the index operation. This operative approach yielded optimal results in our case.

## Introduction

Subtrochanteric fractures account for 10-34% of all hip fractures [1] and exhibit a bimodal distribution, occurring as high-energy injuries in younger patients and low-energy injuries in the elderly [2]. The variation in the reported incidence reflects inconsistencies in fracture classification, as multiple systems have been described in the literature [3]. Intramedullary (IM) nailing has long been established as the gold standard for treatment [4]. However, regional deforming forces and the comminution often seen in high-energy trauma can easily compromise the quality of reduction [5].

In IM nailing, malreduction may present as leg-length discrepancy, rotational deformity, or angular deformity (particularly varus) [1,6]. Because rotational malreduction is common, several techniques have been described to minimize intraoperative errors [6]. Cases that heal with persistent rotational deformity are typically managed with implant removal followed by derotational osteotomy. We report a case of subtrochanteric fracture malreduction that was treated with an open femoral derotational osteotomy performed around the retained nail, without inserting new fixation hardware.

## Case presentation

**Figure 1:**
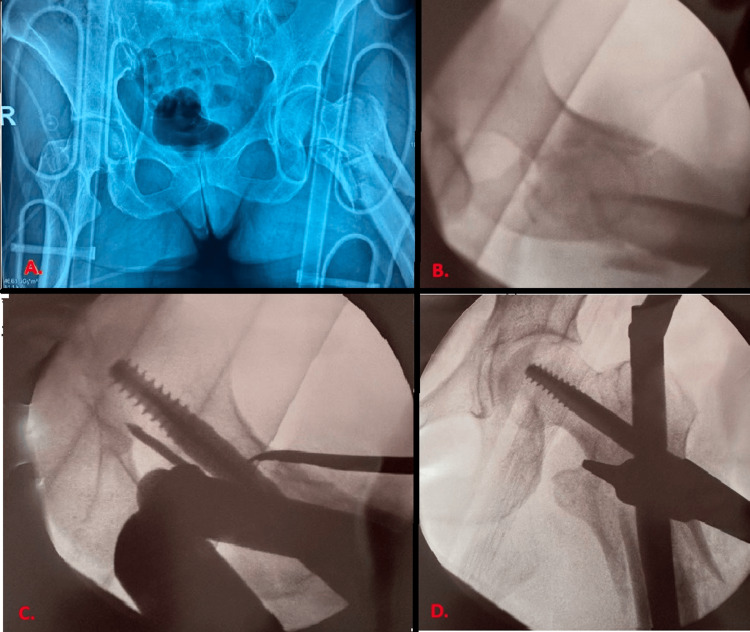
Preoperative and intraoperative radiographs (A) AP radiograph of the pelvis at presentation in the emergency room. (B) Intraoperative C-arm image of the fracture under traction at the start of the index operation. (C, D) Lateral and AP intraoperative C-arm radiographs, respectively, showing femoral neck reduction with a Hohmann retractor and hip screw placement during the same operation. A Steinmann pin was used to prevent femoral head rotation during hip screw insertion.

A 47-year-old woman was admitted to our Orthopedic Clinic (Level I Trauma Center) following a motor vehicle accident. Her past medical history was unremarkable. The patient sustained a skull base fracture, a left sacral fracture, a left humeral transverse diaphyseal fracture, and a comminuted left subtrochanteric fracture (AO/OTA 31-A2.3) (Figure 1). A neurosurgical consultation was obtained, and conservative management was chosen for the skull fracture. The patient’s Injury Severity Score was calculated at 22; therefore, definitive fixation was planned: IM nailing for the femoral fracture and open reduction and internal fixation with a plate for the humeral fracture. The sacral fracture was managed conservatively.

Both extremity fractures were operated on two days later, beginning with fixation of the subtrochanteric fracture using a 240-mm G nail. The patient was placed supine on a traction table. The femoral neck was percutaneously reduced using a Hohmann retractor (Figure 1). The reduction was deemed adequate on the intraoperative lateral radiograph. However, in the AP view, there was significant medial cortical comminution; thus, cortical thickness served as the only reliable landmark for reduction. The left humeral fracture was subsequently treated through an anterolateral approach. The postoperative course was uneventful, and the patient was discharged four days later. There was no apparent intoeing of the operated limb, and the patient did not report any related complaints.

**Figure 2:**
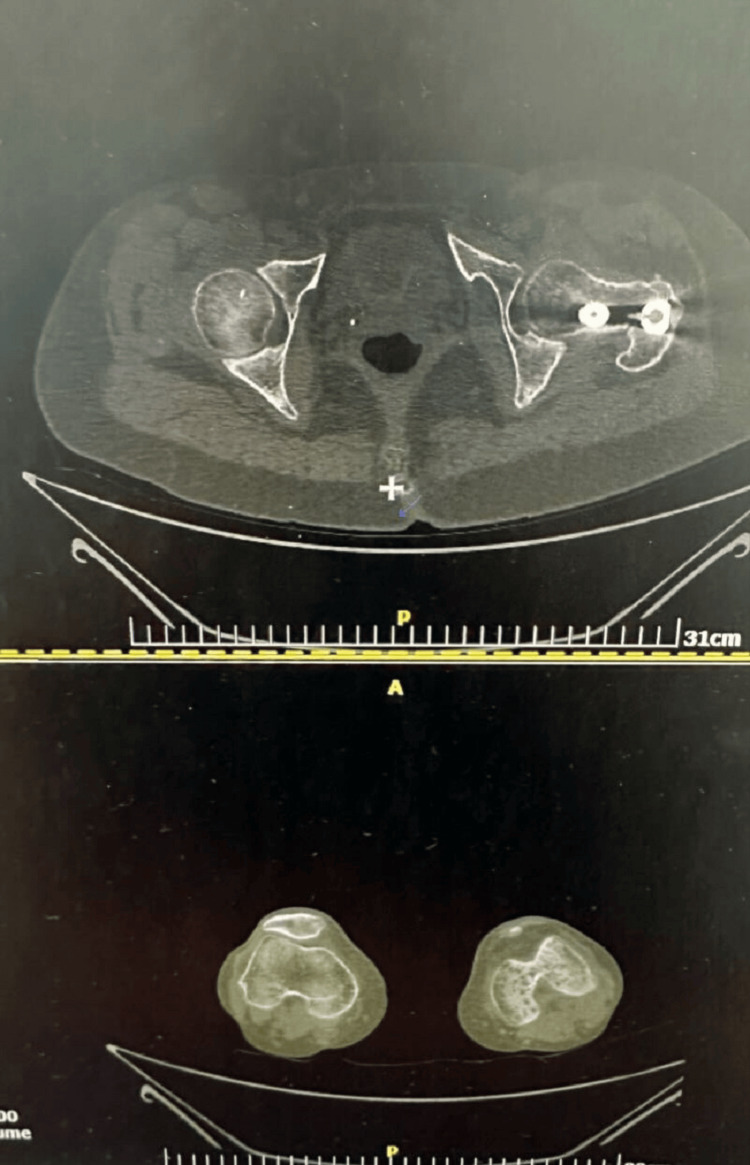
AV measurement Axial images from the postoperative CT scan used to calculate the femoral AV angle. AV, anteversion

At the 12-week follow-up, the patient reported knee pain and a subjective sensation of leg-length discrepancy. Clinical examination revealed a marked limitation in external rotation of the left hip. This finding was initially attributed to delayed weight bearing and ambulation due to the sacral fracture. Radiographs demonstrated callus formation without evidence of heterotopic ossification. A lower limb scanogram showed no leg-length discrepancy; however, CT revealed a 30° internal rotation difference compared with the contralateral femur (Figure 2). Notably, the anteversion (AV) angle of the contralateral leg was approximately 0°.

**Figure 3:**
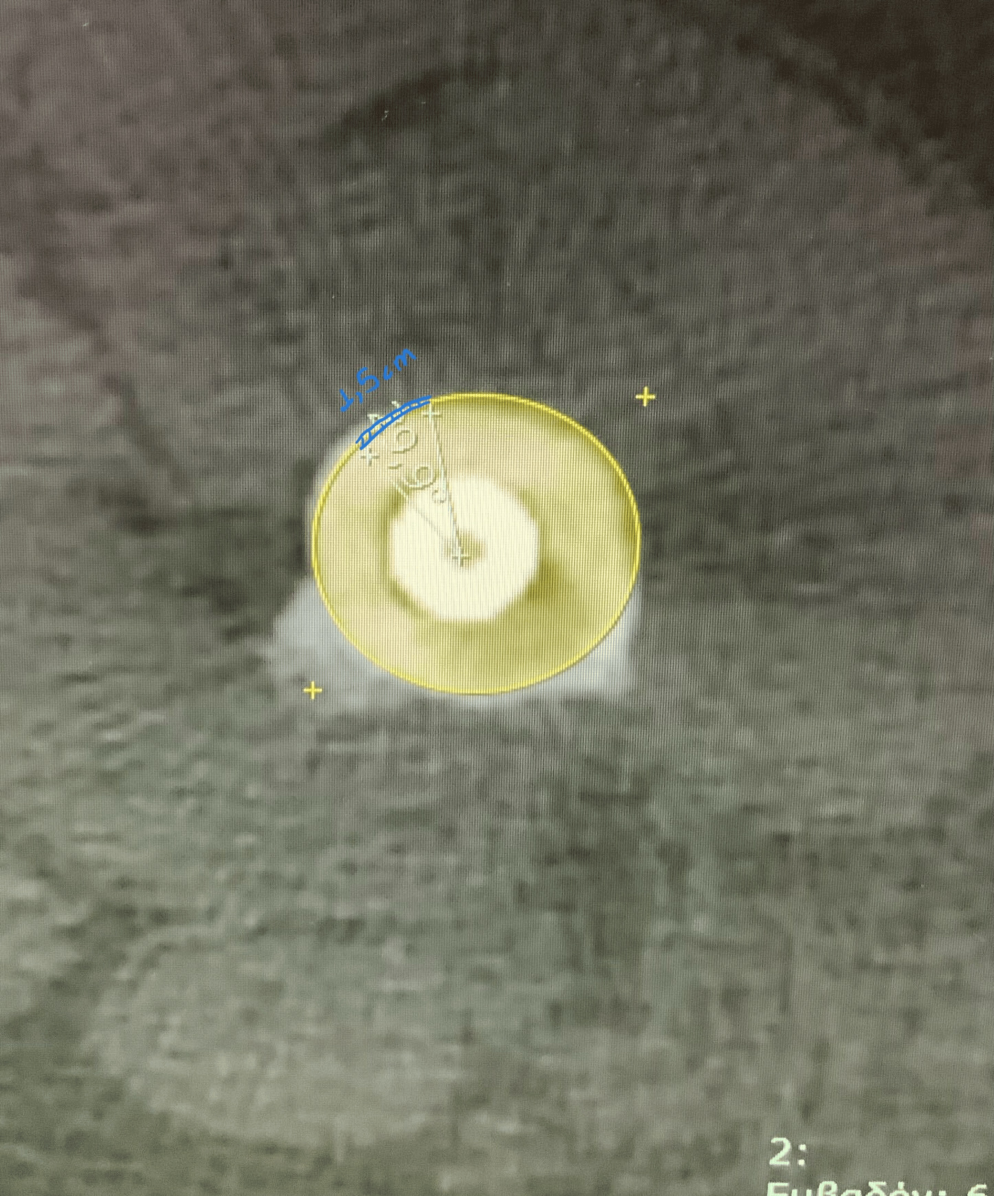
Preoperative planning Axial image from the same CT scan showing the 30° angle measured at the osteotomy level and the corresponding translational distance.

Corrective surgery was proposed, and the patient provided informed consent. The femoral radius at the planned osteotomy level was calculated using CT scan images. To achieve a 30° correction, the corresponding arc length was determined to be 1.5 cm of external rotation. A random point on the circumference was marked with electrocautery using a straight line, followed by a second parallel line marked 1.5 cm ventrally. The principle was that, when these two lines aligned, a 30° derotation would be achieved (Figure 3).

The patient was positioned supine on a radiolucent traction table. The ipsilateral foot was secured in the traction boot with the patella facing upward, and the contralateral leg was placed in a lithotomy position to facilitate C-arm imaging. A lateral incision was made, and the tissues were dissected using a vastus-splitting approach until the femur was adequately exposed.

**Figure 4:**
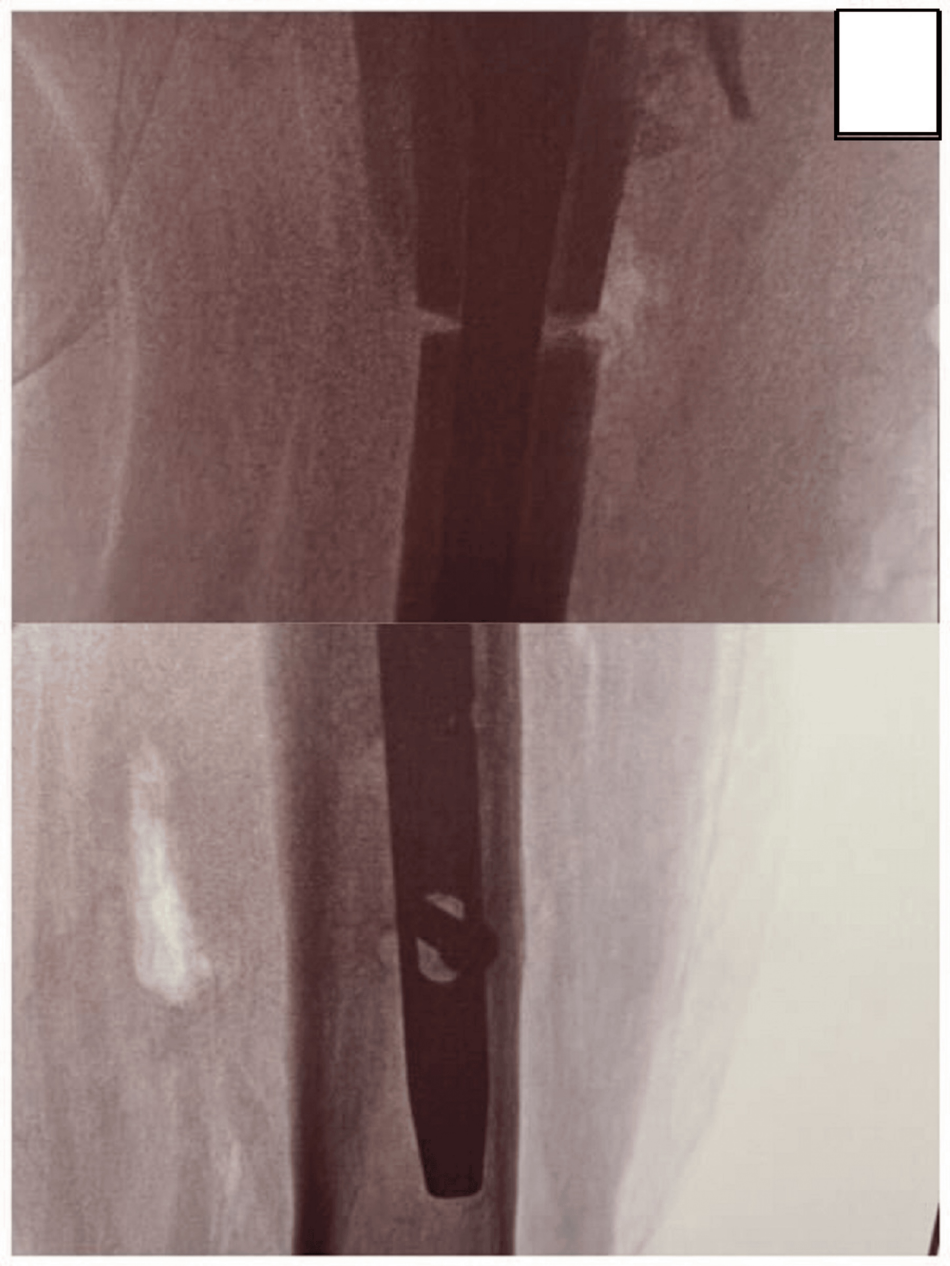
Intraoperative radiographs Intraoperative C-arm images from the second operation showing the femoral osteotomy (AP view, above) and the newly placed distal interlocking screw (lateral view, below).

After confirming the osteotomy level with the C-arm, a Gigli saw was passed over the femur using a Deschamps carrier. With a back-and-forth motion, the osteotomy was performed circumferentially around the nail without causing damage. The distal interlocking screws were then removed through small stab incisions to allow derotation of the distal femur. Once the osteotomy was complete, the distal segment was externally rotated by an assistant on the traction table until the two preoperative reference lines were aligned. The distal interlocking screws were then placed freehand; due to the significant correction required, the screws were inserted in entirely new holes, avoiding overlap with the previous drillings. Standard wound closure was performed, and intraoperative radiographs confirmed optimal positioning of the distal screws (Figure 4).

**Figure 5:**
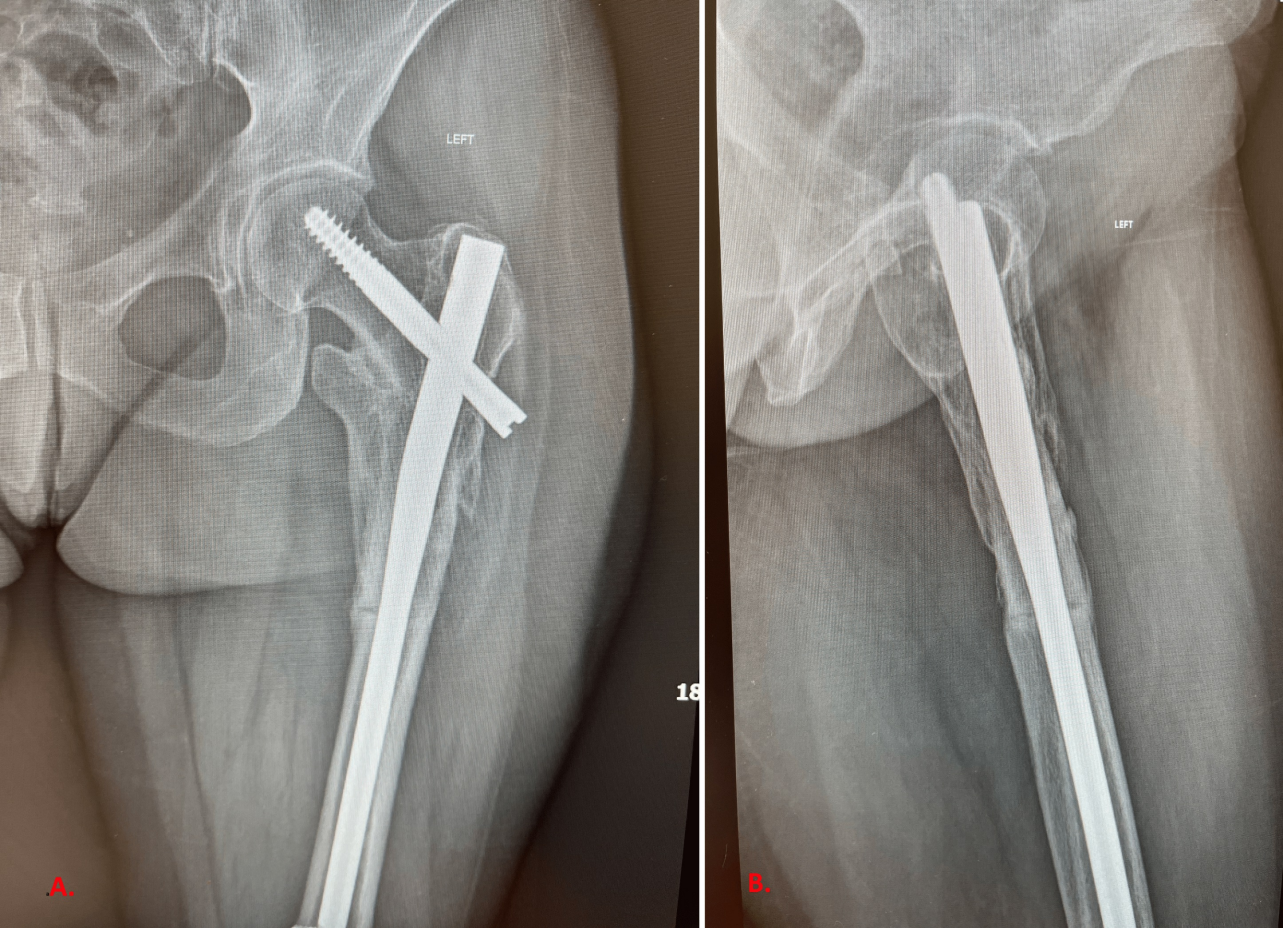
Follow-up radiographs (A, B) Left hip AP and lateral radiographs, respectively, obtained at the four-month follow-up visit.

The patient was discharged on the second postoperative day and advised to begin early weight bearing as tolerated. The early postoperative course was uneventful, with radiographs at four months showing callus formation (Figure 5). The Harris Hip Score was 77.9% and 93% at the sixth and 12th postoperative months, respectively. Knee pain resolved by six months; however, mild limitation in external hip rotation and a slight limp persisted at 12 months.

## Discussion

We describe a case of a subtrochanteric fracture treated with an IM nail that was locked in excessive internal rotation. The patient underwent an open subtrochanteric osteotomy without nail exchange, performed well after fracture consolidation.

IM nailing has long been considered the gold standard for treating subtrochanteric and femoral shaft fractures, offering several advantages, including early mobilization, high union rates, and minimal surgical exposure and dissection [7,8]. However, closed reduction carries a risk of malreduction. Several studies have reported high rates of rotational malalignment after IM nailing of femoral fractures, ranging from 8% to 56%, with higher rates observed in cases with severe comminution [7,9-12].

The average rotational difference between limbs can reach up to 15° in healthy individuals [13]. Consequently, mild postoperative rotational deformities of less than 15° are generally well tolerated clinically [14]. Patients in this cohort can typically compensate during activities such as standing and walking, but may be limited in more demanding tasks [15]. Although internal rotation is often tolerated [8], vigorous activities such as running or sports can result in significant clinical symptoms, particularly when compared with external rotation malreduction [9]. In addition to persistent hip pain, femoral rotational deformity may alter knee joint orientation and the mechanical axis of the limb, potentially accelerating degenerative changes and leading to osteoarthritis [16]. Other studies suggest that malrotation causes eccentric knee joint loading, predisposing to joint degeneration and functional impairment [17]. These findings are consistent with our patient’s clinical presentation, which included knee pain and reduced hip range of motion.

Achieving intraoperative rotational alignment in subtrochanteric fractures can be challenging. Common clinical markers, such as limb appearance, patella position, and soft tissue orientation, are generally unreliable [18-20]. The radiological cortical step sign (CCS) is based on the asymmetry of cortical thickness in the femur; any rotational mismatch between fracture fragments produces a measurable width difference in the apposed cortices on C-arm imaging [20]. Langer et al. validated this concept in a cadaveric study, noting that it is most useful for proximal and middle femoral fractures, and that the direction of malrotation cannot be determined when using the sign as a malreduction marker [18]. Fang et al. further investigated the CCS, concluding it can be helpful in select circumstances [20]. In our case, severe subtrochanteric comminution precluded the use of the CCS, which we believe contributed to the postoperative malreduction.

Computer-assisted navigation for IM nailing is increasingly reported in the literature [21,22]. Its potential benefits in improving fracture reduction and reducing reoperation rates must be balanced against increased cost and longer operative time. Navigation can enhance anatomical reduction compared with freehand distal locking, although availability is limited [23]. Several methods have also been proposed for rotational alignment assessment by comparing the anatomy of the injured and contralateral femurs. Deshmukh et al. described using the contralateral lesser trochanter profile via C-arm imaging to assess rotation [19], a method supported by an in vitro study by Jaarsma et al. [24]. Tornetta et al. described using the C-arm to determine femoral version (FV) of the uninjured limb [25]. However, these techniques often require patient repositioning or result in increased radiological exposure. In 2014, Espinoza et al. proposed using the inherent AV (11-12°) of modern antegrade nails to fix the injured femur and prevent malrotation. This technique avoids evaluation of the contralateral femur and can be applied in cases of severe comminution that prevent cortical apposition [26]. This approach does not account for potential nail-induced deformation, although current solid nails rarely produce significant rotational deformity [27].

In malreduced cases, rotational error is assessed by measuring FV and comparing it with the contralateral side. Jaarsma et al. defined FV as the angle between a line tangential to the posterior femoral condyles and the femoral neck axis [28]. Adult FV averages 14°, but ranges from 4° to 20° [29]. CT is the most accurate method for FV measurement, independent of operator or patient positioning [28,29]. In our case, CT was used to determine the FV difference between sides and calculate the arc length needed for correction. Navadgi et al. applied a similar calculation to correct length and rotational deformities in seven patients [30]; however, they did not report the FV of the contralateral femurs. Our patient had a borderline normal FV of 0°, meaning the “usual” 15° reduction would have resulted in rotational malreduction.

Various methods exist to correct femoral rotational deformity after IM nailing, depending on whether the fracture has united. A derotation osteotomy is indicated if malrotation is identified after fracture healing. Closed osteotomy using IM saws has been employed for derotation and shortening procedures [31]. Itoman et al. developed an IM saw for post-nailing derotation osteotomies and reported excellent outcomes in ten patients [32]. Advantages of a closed osteotomy include lower non-union and infection rates due to minimal dissection [7,32].

Similar to our case, Jagernauth et al. used a Gigli saw for an open osteotomy to correct a 30° malrotated femoral shaft fracture previously treated with an IM nail six months earlier [33]. Unlike our method, they used intraoperative K-wires to achieve the desired derotation. Sloan et al. reported a similar approach to correct a 46° external rotation deformity in a midshaft femur one week post-nailing [34]. However, accurate measurement can be difficult using these techniques because K-wires may not be level, and goniometer readings can be compromised. For this reason, we preoperatively calculated the desired correction using point arc translation rather than direct angulation measurement.

Van Genechten et al. reported subtrochanteric fracture derotation using imaging software to create a patient-specific 3D-printed guide for eccentric K-wire placement [35]. The osteotomy was performed with an oscillating saw over a provisional nail, later exchanged. K-wires were placed unicortically to avoid interference with the nail. In 2023, Goethals et al. described the correction of 19 malrotated femurs post-IM nailing. Low-dose CT was performed postoperatively, and Schanz pins were used to rotate the fragments with the original nail in place. Rotation was visualized with pins perpendicular to the floor, and a digital goniometer (Bonesetter app) was used to measure correction [12]. This represents the first reported use of a digital application for evaluating rotational correction. Limitations of these methods include increased cost and time required for guide fabrication.

A summary of published studies describing various femoral derotation procedures is presented in Table 1.

**Table 1:** Studies detailing femoral derotation procedures included in our literature review AV, anteversion; ER, external rotation; IM, intramedullary; IR, internal rotation; pt, patient; ROM, range of motion

Study	N	Age (mean)	Male/female ratio	Original injury	Deformity (type/degrees)	Surgical technique	Level of osteotomy	Mean time after index operation	Follow-up (months)	Outcome
Stahl et al. (2006) [7]	14	42 (18-63)	6	N/A	11 pts ER mean: 39.5° (26-64); three pts IR mean: 33.3° (29-37)	Closed IM sawing/IM nail; K-wires at femoral condyle and neck	At the thinnest diameter of the femur	47 (five to 132) months	42 (24-74)	Mean postoperative deformity: 0.7°; one infection, two delayed unions
Kent et al. (2010) [10]	2	30 (15-45)	Two males	Mid-shaft femoral fracture	IR mean: 32.5° (30-35)	Open callus removal; nail retention with new interlocking screws; K-wires on either side of the fracture	Fracture site	One pt: seven days; one pt: two months	6	Union, no complications reported
Ahrend et al. (2021) [11]	1	60	N/A	Subtrochanteric fracture	ER: 24°	Schanz screws placed at the desired angle at the femoral neck and distal femur (angled with a triangle); open callus removal; nail exchange	Fracture site	1.5 months	1.5	3° IR compared to contralateral femur; no complications reported
Goethals et al. (2023) [12]	19	30.1 (18-55)	N/A	Comminuted femoral shaft fractures (nine midshaft, four proximal, and six distal third)	Mean malrotation: 24° (18-47)	Closed; four Schanz pins (two for reference for angle calculation and two for derotation maneuver); digital app for angle calculation; nail retention with new interlocking screw insertion	No osteotomy	Within three days	N/A	Mean correction to 4° (0-8°) compared to the contralateral side
Navadgi et al. (2004) [30]	7	39 (22-60)	6	Femoral shaft fracture	Malrotation: 21° (10-30) and shortening: 27 mm (20-43)	Open peg (or step) type femoral osteotomy with Gigli saw under cortico-periosteal sleeve; derotation using point of arc translation; lengthening; IM nail retention	N/A	180 (24-432) months	5.7 (4-8)	Residual malrotation mean: 5° (0-14); one delayed union requiring nail dynamization; one pt with breakage of distal interlocking screws
Chapman et al. (1993) [31]	37 (six for derotation)	30 (16-68)	2.36	31 femoral shaft fractures, one excessive femoral AV, four congenital deformities, one myelodysplasia, one childhood osteomyelitis, and one poliomyelitis	Five pts ER: 58°, one pt IR: 85°, others leg-length discrepancy mean: 3.3 cm	Closed femoral osteotomy with IM saw and back-cutting osteotomes; IM nailing (locked and unlocked)	N/A	N/A	39.6 (12-132)	All pts regained preoperative ROM; pts with unlocked nail had no postoperative mobilization; antirotation boot used; derotation: all pts within 5° of the contralateral extremity; two pts hip abductor weakness at one year, treated with nail removal
Itoman et al. (1996) [32]	8	25 (18-53)	7	Femoral shaft fracture (six comminuted, two transverse)	ER mean: 46° (40-60)	Closed femoral osteotomy with IM saw; angled Steinmann pins in condyle and neck; IM nailing	Isthmus of the medullary canal	N/A	12-60	Mean postoperative malrotation: 1.25° (0-5); not specified for femoral derotation (10 pts total in cohort, two with tibial derotation); one delayed union conservatively treated; two pts slight discomfort after long walks
Jagernauth et al. (2012) [33]	1	19	N/A	Spiral femoral shaft fracture	IR: 30°	Angled K-wires at either side of the fracture (using tibial osteotomy protractor); open femoral osteotomy with Gigli saw; bone graft; nail retention; new interlocking screws	5 cm proximal to distal locking screws	Six months	6	Osteotomy site union; pain-free, normal gait
Sloan et al. (2020) [34]	1	23	N/A	Midshaft femoral fracture	ER: 44°	Parallel Schanz pins at neck and distal femur with sterile goniometer; nail retention with new distal interlocking screws	No osteotomy	Seven days	N/A	5° ER compared to the contralateral side
Van Genechten et al. (2023) [35]	1	32	N/A	Subtrochanteric fracture	IR: 36°	Unicortical angled K-wire placement using 3D-printed patient-specific guide; nail removal and provisional nail insertion; open osteotomy with oscillating saw; nail exchange; same lag screw path with new distal interlocking screw insertion	Subtrochanteric level	Three months	6	0.1° difference in AV compared to the contralateral side; union at four months; lag screw and proximal nail irritation at six months, treated with hardware removal

## Conclusions

Rotational deformities in subtrochanteric fractures are relatively common. After fracture consolidation, open derotation osteotomy using a Gigli saw without removing the nail is a safe and effective treatment, allowing correction of severe deformities with minimal limb-length loss, as demonstrated in our patient. Potential pitfalls of this technique include overlapping distal screw drillings from prior fixation and the risk of nail damage with aggressive sawing. Although no complications were observed in our case, further studies are needed to fully evaluate the safety and efficacy of this approach.
